# Silver-Nanocellulose Composite Used as SERS Substrate for Detecting Carbendazim

**DOI:** 10.3390/nano9030355

**Published:** 2019-03-04

**Authors:** Luqiang Huang, Changji Wu, Lijuan Xie, Xue Yuan, Xinyu Wei, Qun Huang, Youqiang Chen, Yudong Lu

**Affiliations:** 1 College of Life Sciences, The Public Service Platform for Industrialization Development Technology of Marine Biological Medicine and Product of State Oceanic Administration, Southern Institute of Oceanography, Fujian Normal University, Fuzhou 350117, China; biohlq@fjnu.edu.cn (L.H.); 18060618124@163.com (L.X.); yuanxue89@hotmail.com (X.Y.); yqchen@fjnu.edu.cn (Y.C.); 2 College of Chemistry and Materials Science, Fujian Provincial Key Laboratory of Advanced Materials Oriented Chemical Engineering, Fujian Key Laboratory of Polymer Materials, Fujian Normal University, Fuzhou 350007, China; 18060489009@163.com (C.W.); 18750781690@163.com (X.W.); 3 Fujian Provincial Key Laboratory of Quality Science and Processing Technology in Special Starch, Fuzhou 350002, China; huangqunlaoshi@126.com

**Keywords:** nanocellulose, surface enhancement Raman scattering (SERS), carbendazim, Ag nanoparticle

## Abstract

Nanocellulose is an abundant green resource that, owing to the larger surface area, length, and diameter of the fibers, can be used as a framework for loading Ag nanoparticles and serve as substrate for surface enhancement Raman scattering (SERS). These properties would cause the hydroxyl groups on the surface to adsorb the Ag ions and reduce them to Ag seed to form a load fulcrum. This paper presents a convenient and environmentally friendly method for the fabrication of silver-nanocellulose composites (NCF-Ag). A commonly used pesticide, carbendazim (CBZ), was used as a SERS probe to evaluate the properties of NCF-Ag. The results showed that NCF-Ag possesses good homogeneity, reproducibility, and stability. Additionally, CBZ was found to have a low limit of detection (LOD), i.e., 1.0 × 10^−8^ M, which indicates the possibility for trace analysis. Furthermore, it presents good linearity with *R*^2^ = 0.98 at 1007 and 1270 cm^−1^ in the range from 10^−4^~10^−7^ M CBZ.

## 1. Introduction

Carbendazim (CBZ), owing to its high insecticidal activity, is reported to be one of the most commonly detected insecticides, and is widely used to control the various fungal diseases of agricultural crops. Farmers tend to use excess amounts of pesticides to ensure the diseases are fully under control and to protect the crops. For example, researchers examined pesticide residues in 50 banana samples imported into Italy and found that five samples contained carbendazim, two of which exceeded the FAO and WHO Codex Alimentarius and the maximum residue limits prescribed by the Italian Department of Health [1]. Other than this, carbendazim residues were detected in 1674 out of 20,496 samples of more than 24 types of vegetables, collected between 2014 and 2016 throughout China [2]. Although CBZ is of lower toxicity to mammals, higher doses would damage their reproduction and growth [3]. However, the half-life of carbendazim varies from several days to a few months in water and soil [4] and this may have an impact on the aquatic organisms and soil biodiversity. In addition, it may be harmful to humans. Several studies have revealed toxic effects of carbendazim on spermatogenesis induction [5], female reproduction and fetal development [6]. Thus, CBZ was studied in more detail. Previous research reported that acute toxicity results showed 8.6 mg/kg dry soil CBZ could significantly induce DNA damage to the earthworm [7], and some aquatic organisms such as milk fish [8], zebrafish embryos [9], zebrafish larvae development [10], and human placental trophoblast cells [11] have been studied. These studies showed that carbendazim is harmful to organisms in that it induces multiple responses including oxidative stress, apoptosis pathway, immune and lipid peroxidation, acetylcholine esterase, and interferes with the endocrine systems. Thus, a number of methods to detect CBZ such as Ultraviolet-vis spectroscopy [12], HPLC-UV (High performance liquid chromatography with ultraviolet-vis spectroscopy) [13], voltammetry [14], molecular imprinting [15], and surface enhancement Raman scattering (SERS) [16,17] have been improved.

SERS [18] is a selective, highly sensitive, rapid detection method that has the ability to provide abundant information about organic compounds [19]. Metal colloids such as Au [20], Ag [21] or Au@Ag [22] colloids are the most commonly used SERS substrates and play a key role in enhancing its performance. Researchers have been focusing on the use of low cost, green synthesis methods and this objective has also influenced their approach to using SERS. Thus, polymeric materials such as PVP [23] and chitosan [24] were used as template or carrier to prepare the SERS substrate and good results were obtained. Cellulose, an abundant global resource, has found use as material in many applications. Lu et al. [25] used modified cellulose as a stabilizer and protective agent for an Ag colloid and obtained good SERS performance. A paper-based substrate has been popular in recent years, not only because it is inexpensive, but because of the natural wrinkles and fibril structures of paper. These structures allow metal nanoparticles to be deposited and arranged on paper to form large-area SERS “hot-spots” [26,27]. Francis et al. [28] used hydroxypropyl cellulose as a template to synthesize Ag NPs and demonstrated that Ag^+^ readily combines with O^−^ in the R-OH or ROOH groups of cellulose, which provide us with attachment sites. In our previous work [29], we used cellulose to adsorb the Ag^+^ and used the reducibility itself to form Ag seeds and shaped Ag-coated cellulose fibers in the presence of the hydroxylamine hydrochloride. Limei Tian et al. thought that the bacterial nanocellulose (BNC) film base could obtain significantly lower surface roughness than conventional filter paper consisting of normal cellulose, so they made the BNC film and fabricated and loaded Au nanoparticles by a gravity­assisted filtration method as a SERS substrate [30]. Xinchang Pang et al. [31] used the modified cellulose as a nanoreactors to prepare various nano-size particles. Compared with normal cellulose, the width of nanocellulose fibers is in the nanometer range, thereby providing a much larger surface area on which to load metal nanostructures [32]. The advantage of nanocellulose could lead to higher sensitivity and better homogeneity as a SERS substrate, and it could form a rigid structure to induce the growth of Ag clusters. Compared to the existent nanocellulose-based SERS substrate in the literature [26,27], the NCF-Ag prepared in this work is reduced Ag particles in situ, offered larger density Ag particles with good uniform. Moreover, the preparation process is simpler and more convenient.

**Figure 1:**
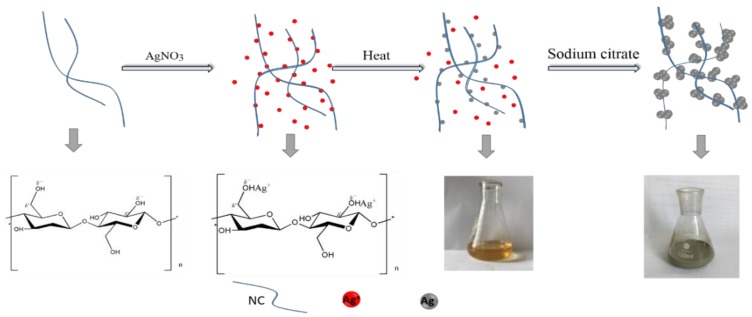
Mechanism and process for the fabrication of the substrate consisting of NCF-Ag.

In this work, a silver-nanocellulose (NC) composite was prepared by using a one-step and environmentally friendly method in an aqueous system. As illustrated in Figure 1, Ag^+^ was first adsorbed onto NC by hydroxyl, after which Ag seeds were formed on the surface of the NC as fulcrums. Then, sodium citrate was added as a reducing agent and lastly, Ag seeds were grown in the presence of excess Ag^+^.

## 2. Material and Methods

### 2.1. Reagents and Aparatus

Nanocellulose fiber (NCF) powder was provided by Shanghai Shansi Nanotechnology Co., LTD. (Shanghai, China). Silver nitrate (purity ≥ 99.8%), sodium citrate dehydrate (purity ≥ 99.8%), and carbendazim (CBZ) were obtained from Shanghai Aladdin Bio-Chem Technology Co., LTD(Shanghai, China). Aluminum metal sheet was obtained from Sinopharm (Shanghai, China). Analyses were conducted using UV-Vis spectrometry (UV 1902, Lengguang Tech., Shanghai, China) and scanning electron microscopy (SEM) (Regulus 8100, HITACHI, Tokyo, Japan).

All solutions except carbendazim solution were prepared using Millipore water (Guozhiyuan Y1/2-10UV Kertone, Changsha, China) and carbendazim solution was prepared with absolute ethanol.

### 2.2. Preparation of NCF-Ag

The NCF-Ag was fabricated by adding 10 mL silver nitrate solution of different concentrations (3, 5, 8, and 10 mM) to 10 mL of 0.4% NCF suspended in solution, and the suspension solution was stirred slowly for 30 min to ensure that the silver ions were well adsorbed onto the surface of the NCF. Next, the suspension was heated and boiled until its color became orange and Ag seeds formed on the surface of the NCF, after which 10 mL of 1 wt % sodium citrate solution was added under vigorous stirring. Subsequently, boiling was continued for 10 min to allow the NCF-Ag NPs to mature. The pH of NCF-Ag suspension solution was eight.

### 2.3. Sample Preparation

Then, 3 μL carbendazim solutions of different concentrations were added to 3 μL NCF-Ag NPs sediment and the suspension was thoroughly mixed for approximately 5 min. Then, the mixture was transferred to the aluminum sheet and allowed to dry at room temperature.

### 2.4. Measurement

SERS spectra were acquired by a Renishaw confocal Raman instrument (London, UK) equipped with a 785 nm laser and 20× objective. The accumulation time for each measurement was 10 s. Two samples were tested and 5 spectra were taken from each sample for every spectrum.

### 2.5. Data Analysis

A Vancouver Raman algorithm based on a fifth-order polynomial fitting method [33] was used to remove fluorescence background and noise signals for all the raw SERS spectra. In addition, Origin 8 software (OriginLab, Hampton, MA, USA) was used to analyze the data.

## 3. Results and Discussion

**Figure 2:**
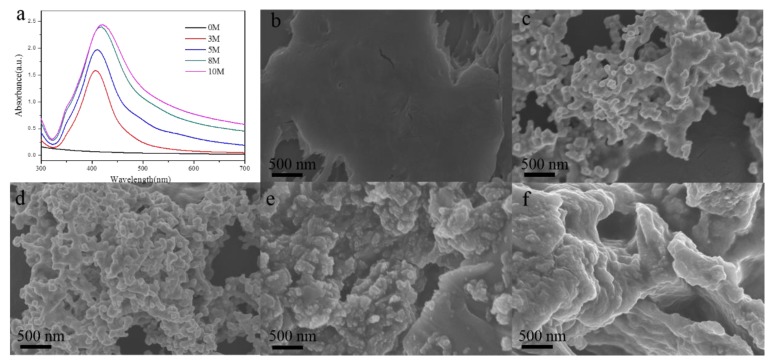
(**a**) UV-vis adsorption spectra of NCF (Black), NCF-Ag-3 mM (Bright red), NCF-Ag-5 mM (Blue), NCF-Ag-8 mM (Green), and NCF-Ag-10 mM (Plum purple); (SEM) images of (**b**) NCF, (**c**) NCF-Ag-3, (**d**)NCF-Ag-5, (**e**) NCF-Ag-8, (**f**) and NCF-Ag-10.

**Figure 3:**
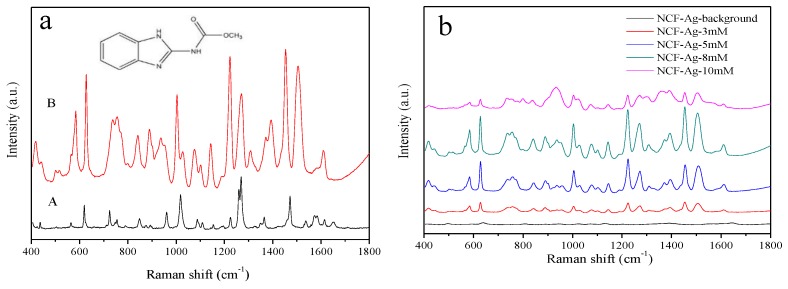
(**a**) Spectra of carbendazim (CBZ): (A) Raman spectrum and (B) SERS spectrum of 10^−4^ M CBZ detected with NCF-Ag NPs prepared with 8 mM AgNO_3_. (**b**) SERS spectrum of the NCF-Ag NPs with 8 mM AgNO_3_ and SERS spectra of 10^−4^ M CBZ detected with NCF-Ag NPs prepared with 3, 5, 8, and 10 mM AgNO_3_.

The as-prepared NCF-Ag NPs in solutions with different silver nitrate concentrations (3, 5, 8, 10 mM) were characterized by UV-vis spectroscopy and SEM. Figure 2a shows that the plasmon spectrum of NCF-Ag NPs clearly presents maximum absorption peaks at 407, 410, 417, and 420 nm. Its red shifting should be caused by the enlarged NCF-Ag or its aggregation by the concentration of silver ions increased. Similar to the UV-vis spectra, the SEM images in Figure 2c–f show that NCF-Ag was induced to grow in the form of the branches at low concentrations of Ag ions, and that aggregation increased to form blooms (e to f) in the presence of high concentrations of Ag ions. We used 10^−4^ M CBZ as a probe to test the SERS ability of NCF-Ag. The Raman spectrum of CBZ and the SERS spectrum of the CBZ are shown in Figure 3a. Their characteristic peaks are listed in Table 1. Compared to the Raman spectrum, the bands present in the SERS spectrum of CBZ were slightly shifted, which could be attributed to the strong polarization that occurred at the surface of the NCF-Ag NPs. The N–H bending modes of the benzimidazole ring is shifted downward from 1260 to 1224 cm^−1^, 1473 to 1460 cm^−1^ and greatly enhanced, indicating a head-on close to the vertical orientation of NCF-Ag NPs on the surface, which is similar to the Sun’s study [34]. In addition, the SERS spectra exhibit clear and distinct peaks. The intensity of the SERS band at 1224 cm^−1^ is higher than that at 1270 cm^−1^, contrary to the Raman spectrum. The result (pH = 8) agrees with Furini’s work (pH = 6–10) [16] in that the 1224 cm^−1^ band, attributed to amide, decreases when the pH increases, whereas the 1270 cm^−1^ band belonging to imidazole intensifies. In the pH range from 6–10, the CBZ adsorbed on Ag NPs was the modality of CBZ^0^–Ag^+^ (imidazole(N)-Ag) and the N–H band occurred in the same plane. Outside of this pH range, the adsorption modality would change and the ratio of 1224 cm^−1^/1270 cm^−1^ changes too.

SERS spectra of 10^−4^ M CBZ with NCF-Ag are shown in Figure 3b to further demonstrate the optimal performance of NCF-Ag. The intensity of CBZ of the former increases and then decreases and NCF-Ag-8 shows the strongest enhancement. This is consistent with the results inferred from the SEM images of the NCF-Ag NPs. As the Ag branches increasingly developed blooms, the SERS hotspot increased; however, some analytes adsorbed under the branch and remained undetected by the laser. These branches were unable to contribute to the SERS intensity as in our previous work [29]. When the Ag ion concentration reached 8 mM, the compactness of Ag branches intensified. This reduced the undetected analyte and led to the growth of many small “spheres” on the surface to form nanogaps and augment the number of “hotspots”. When the Ag^+^ concentration reached 10 mM, super abundancy resulted in the Ag branches becoming overgrown and massive, causing the nanogaps in NCF-Ag-8 to diminish.

**Figure 4:**
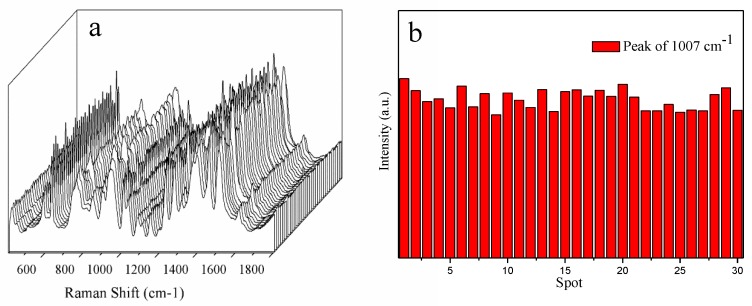
(**a**) SERS spectra of 1.0 × 10^−4^ M CBZ collected at 30 randomly selected spots on the same NCF-Ag NPs. (**b**) Graphs of the signal intensity of the 1007 cm^−1^ line from 1.0 × 10^−4^ M CBZ collected at 30 randomly selected spots on the same NCF-Ag NPs.

**Figure 5:**
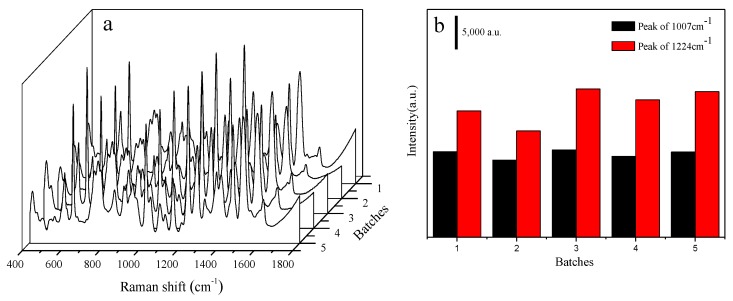
(**a**) SERS spectra of CBZ tested with batches of NCF-Ag-8. (**b**) Bands at 1224 and 1270 cm^−1^.

The homogeneity of NCF-Ag NPs was estimated by randomly sele cting 30 spectra of 10^−4^ M CBZ obtained from one sample of NCF-Ag-8 (Figure 4a), which shows its good uniformity. Additional details are shown in Figure 4b, where the intensity of the characteristic Raman band at 1007 cm^−1^ is a result of C–N bending, C–C stretching, and C–O–CH_3_ stretching and the results indicated good homogeneity with RSD = 7.8%. The reproducibility was also tested by preparing batches of NCF-Ag-8, which were tested with 10^−4^ M CBZ, as shown in Figure 5, resulting in good reproducibility. In other words, the chances of repeating it with RSD = 7% are 80%.

**Figure 6:**
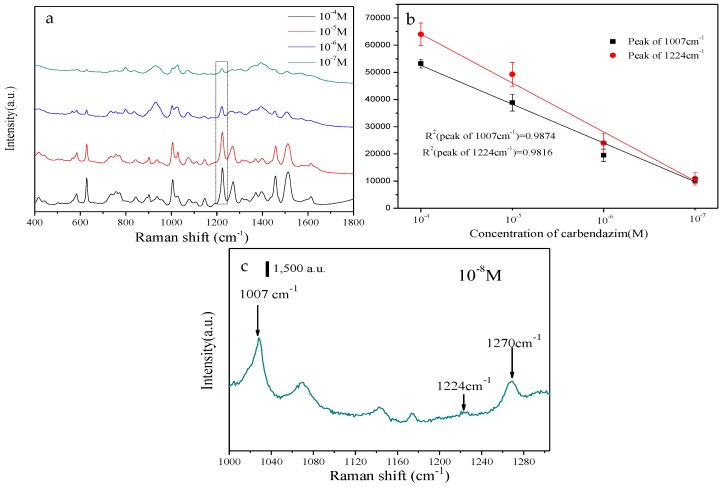
(**a**) SERS spectra of different concentrations of carbendazim. (**b**) Peaks at 1007 and 1224 cm^−1^ on the SERS spectra shown in a. (**c**) SERS spectrum of 10^−8^ M.

The sensitivity of NCF-Ag-8 was further tested by using it to detect CBZ of various concentrations. Figure 6a shows the SERS spectra of different concentrations of carbendazim from 10^−4^~10^−7^ M, with all of them presenting clearly resolved peaks. In addition, the peak intensity was plotted against the concentration in the range from 10^−4^~10^−7^ M in Figure 6b and showed good linearity with *R*^2^ = 0.98. Moreover, the limit of detection (LOD) for NCF–Ag NPs was determined to be 10^−8^ M CBZ (Figure 6c). SERS spectra of 10^−8^ M exhibited peaks with weak intensities on the basis of the characteristic bands at 1007, 1224, and 1270 cm^−1^. These bands are associated with C–N bending, C–C bending, and C–O–CH_3_ stretching, NH_AM_ and NH_IZ_ in plane deformations [16]. According to Aaron [36] and Furini [35], the benzimidazole group interacts with the NCF and Ag surface because of its closer proximity than the aliphatic group and, which is the reason for the intensified bands at 1224 and 1270 cm^−1^. The low LOD indicates the possibility of using NCF-Ag for trace analysis.

## 4. Conclusions

The NCF-Ag was fabricated using a one-step procedure and an environmentally friendly approach. The results showed that the growth of the Ag NPs was reduced by nanocellulose and was affected by the concentration of Ag^+^, the growth mode of which changed from branch to bulk. Because of the regularity of the “paper” obtained by drying NCF-Ag, it exhibited good homogeneity with RSD = 7.8% that was calculated by recording 30 spectra at randomly selected positions. In addition, it possesses good reproducibility of 80% with RSD = 7%. In terms of the analysis of pesticide residues, the limit of detection (LOD) of CBZ was found to be 10^−8^ M. The concentration showed good linearity with *R*^2^ = 0.98 in the range from 10^−4^ to 10^−7^ M, which indicates the possibility for trace analysis. Our study makes a significant contribution to the results reported in the literature because we could detect the analyte at lower concentrations by using a green, low-cost SERS substrate.

## Figures and Tables

**Table 1:** Band assignment of carbendazim [17,35].

Solid Raman	SERS	Vibrational Description
617 cm^−1^	628 cm^−1^	ring stretching and C–C bending
723 cm^−1^	737 cm^−1^	C–C bending and C–O–CH_3_ bending
960 cm^−1^	–	C–H bending
1018 cm^−1^	1007 cm^−1^	C–N bending and C– bending and C–O–CH_3_ stretching
1260 cm^−1^	1224 cm^−1^	C–C bending and N–H bending
1270 cm^−1^	1270 cm^−1^	C–H bending and N–H bending
1473 cm^−1^	1460 cm^−1^	C–H bending and N–H bending
–	1510 cm^−1^	N–H bending and C–N stretch
